# Be prepared! Impact of structured video-assisted coaching on performance in a simulated bleeding exercise during laparoscopic surgery

**DOI:** 10.1007/s00464-024-11173-3

**Published:** 2024-08-26

**Authors:** Dimitrios Chatziisaak, Moritz Sparn, Daniel Krstic, Gabriele Bauci, Réne Warschkow, Walter Brunner, Bruno Schmied, Dieter Hahnloser, Stephan Bischofberger

**Affiliations:** 1 Department of Surgery, Kantonsspital St. Gallen, St. Gallen, Switzerland; 2 Department of Visceral Surgery, University Hospital Lausanne, Lausanne, Switzerland; 3 Paracelsus Medical University, Salzburg, Austria

**Keywords:** Laparoscopic training, GOALS score, Structured video-assisted coaching, Skill acquisition

## Abstract

**Background:**

Bleeding during laparoscopic surgery is stressful and requires immediate efficient management. Skills for complication management are rarely trained. This study aims to investigate the impact of video-assisted coaching on laparoscopic skills acquisition and performance in emergency bleeding situations.

**Methods:**

Participants faced simulated emergency scenarios during laparoscopy involving bleeding management in porcine aorta/kidney specimens. Four sequences were conducted over two days, with a structured video-assisted coaching provided between sequences. Performance was assessed using the Global Operative Assessment of Laparoscopic Skills (GOALS) score. The study involved 27 participants attending the advanced colorectal surgery module at the 40th Annual Davos Course in 2023.

**Results:**

54 video sequences were analyzed. Structured video-assisted coaching improved the GOALS sum score by 0.36 (95%CI: 0.21–0.50, *P* < 0.001) in contrast to simple repetition (0.05 with 95%CI: −0.43 to 0.53, *P* = 0.826). This association was observed for depth of perception (*P* < 0.001), bimanual dexterity (*P* < 0.001), tissue handling (*P* < 0.001), overall performance (*P* < 0.001), and efficiency (*P* < 0.001). Autonomy did not significantly improve (*P* = 0.55). Findings were consistent regardless of age, gender, and overall laparoscopic experience of the participants. However, a weaker effect of structured video-assisted coaching was observed in participants with experience in laparoscopic surgery.

**Conclusion:**

Structured video-assisted coaching improved performance in laparoscopic skills in complex and stress-inducing bleeding scenarios. The findings of this study support the incorporation of video-assisted coaching and complication management exercises into surgical training curricula.

Today, the classical surgical teaching according to the Halsted “master-apprentice model” is insufficient and inefficient for the acquisition of laparoscopic skills [1]. Socioeconomic factors (e.g., work-hour restrictions and work-life balance) and increasing financial pressures in the health care system add pressure to train residents quickly and efficiently [2]. Surgical residents need to learn minimally invasive skills outside of the operating room so that they can subsequently apply them efficiently on patients.

Acquiring and developing laparoscopic skills through simulation training has been shown to be effective regardless of whether simple box trainers or virtual reality simulators were used [3–5]. It has also been shown that these skills can be safely transferred to the operating room [6]. However, skills during stressful emergency situations are rarely trained. Bleeding is one of the most frequent intraoperative adverse events in laparoscopic surgery occurring in 3.3% of all the laparoscopic procedures [7]. Training in such emergency situations is needed.

According to the model of Fitts and Potter, a three-stage motor skill acquisition procedure is required: cognition (understanding the task), integration (comprehend the task), and automation (perform with speed, efficiency, and precision) [8]. An effective guidance and demonstration followed by deliberate practice with feedback is needed in order to succeed in obtaining new skills. However, a professional and expert supervision of a trainer is time consuming, expensive, and not always possible. Structured video-assisted coaching is a possible solution.

The purpose of this study was to analyze the impact of expert-guided video-assisted coaching on performance and safety of a simulated bleeding exercise in laparoscopic skills training.

## Materials and methods

The dataset of the study was obtained during the 40th Annual Davos Course in 2023, an international surgical training course in open, laparoscopic, and robotic abdominal surgery in Davos, Switzerland (www.davoscourse.ch). All study participants attended the advanced course, colorectal module, and gave written informed consent to the study. Demographics and previous surgical experience were recorded with a questionnaire. Data were analyzed anonymously.

**Fig. 1:**
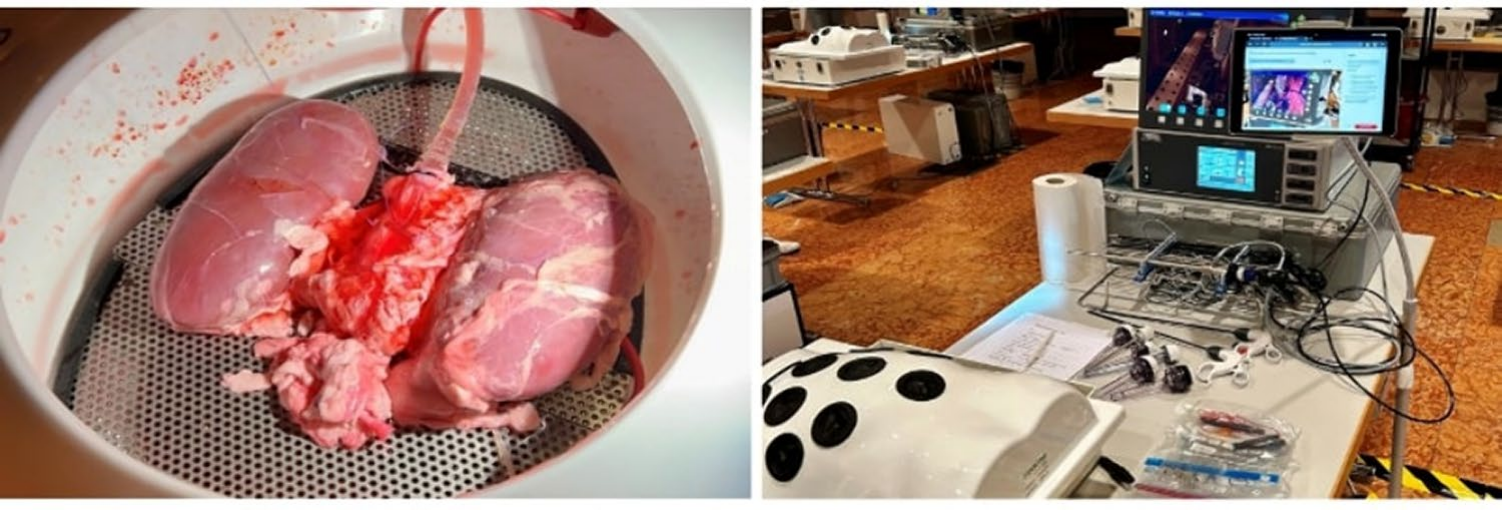
Setting of the perfused porcine model

Each training station was equipped with a complete laparoscopy unit (screen, camera unit, power generator), standard laparoscopic instruments with a focus on hemostasis (clip applicator, linear stapler, different energy devices, needle holder, etc.), and a box trainer with perfused porcine organs (P.O.P Pulsating Organ Perfusion, Optimist GmbH, Innsbruck, Austria). Perfused porcine aorta/kidney specimens that exhibited arterial hemorrhage from a lumbar artery were placed in the box trainers. The participants were given 5 min each to control and effectively stop the bleeding. All exercises were recorded with a commercially available tablet using a prototype web-based application (Simulation AR, Virtamed AG, Zurich, Switzerland) for simultaneous video cloud upload and subsequent video assessment (Fig. 1).

**Fig. 2:**
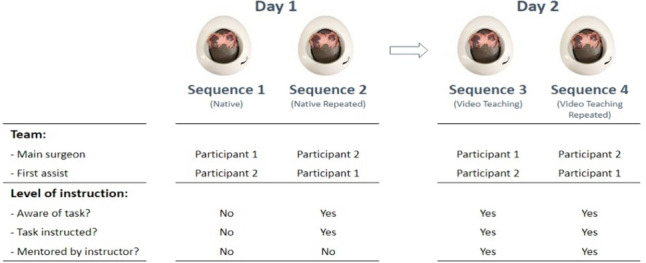
Study sequences

The participants worked in teams of two in the same order on training days 1 and 2. On each day, the same exercise was repeated twice in the team, with each participant working once as the operating surgeon and once as the assistant (Sequences 1–4, Fig. 2). All participants were unaware of the nature of the task they were given at the beginning of the first exercise (Sequence 1, Fig. 2). Bleeding location varied for each exercise. After the first exercise, a detailed structured video-assisted instruction on laparoscopic hemostasis was given: compression techniques, the different instruments available, and hemostasis techniques were explained and demonstrated in detail. All participants watched this video live. Subsequently, the exercise was repeated (Sequence 2, Fig. 2).

On the following day, participants first were given again a structured live video guidance on the different techniques controlling bleeding. In addition, the table instructor provided real-time explanations (but did not interfere with the exercise) (Sequences 3 & 4, Fig. 2). All instructors have previously seen the instruction videos and were trained in the specific exercises (train the trainers). After each sequence, all participants performed a short self-assessment asking the participants to evaluate their performance based on the given task.

After completion of the course, the collected videos were assessed by 7 experienced minimally invasive surgeons using the GOALS score (Global Operative Assessment of Laparoscopic Skills) [9]. The theoretical range of GOALS sum score was 5–25 points. It was scaled to a range of 1–5 for better comparison against its sub-scores. The assessment of the videos was blinded, so that no inference could be made about the participants or the sequence. For every sequence (1–4), the depth of perception, bimanual dexterity, efficiency, tissue handling as well as autonomy were measured as integers on a scale from 1 to 5 and summarized to the GOALS sum score analogical to the results published in the first publication of Vassiliou et al. [9].

Statistical analyses were performed using the recent R statistical software version (www.r-project.org). A two-sided *P* value of less than 0.05 was considered statistically significant. Continuous data are expressed as the mean (standard deviation, SD). In descriptive analysis, proportions were compared by chi-square tests and continuous variables by Mann‒Whitney U tests. Missing values for age (*N* = 2) and gender of the participants (*N* = 1) were imputed with random forests using the R library “randomforestSRC” for multivariable analysis. To address multiple testing and to avoid an inflation of the significance level alpha, Tukey contrasts and their 95% family-wise confidence intervals were estimated and *P* values for the GOALS subscales were adjusted according to Benjamini and Hochberg to control for the false discovery rate (FDR) [10, 11]. Consistency and inter-rater agreement were assessed using the intraclass correlation coefficient (ICC) [12]. ICC values less than 0.5 indicate poor reliability, values between 0.5 and 0.75 indicate moderate reliability, and ICC values exceeding 0.75 indicate good reliability.

The GOALS score and its sub-scores were analyzed using mixed-effects regression analyses with random intercepts for both the reviewers and the probands, allowing for heteroscedasticity in the covariance matrix and were fitted optimizing the maximum likelihood using the R library lme4. The *P* values were estimated by likelihood ratio tests and 95% confidence intervals (CIs) were obtained by the likelihood profile. A Violin plot was used to visualize the treatment effect on the GOALS score simultaneously displaying the probability distribution of the score, its range, and the median with the interquartile range.

## Results

Data of 27 trainees of the advanced course colorectal module (14 male, 12 female, and one participant did not state gender) were analyzed. Participants had comparable previous surgical experience with laparoscopic appendectomies, laparoscopic cholecystectomies, and bariatric surgical procedures as well as experience with hernia and colorectal resection (Table 3).

54 video recordings were analyzed. The Intraclass Correlation Coefficient (ICC) indicate a moderate to good inter-rater reliability for the GOALS sum score (ICC = 0.72, 95%CI: 0.63–0.81, *P* < 0.001). In the sub-scores for the skills assessed by the GOALS sum score, a moderate reliability was observed.

**Fig. 3:**
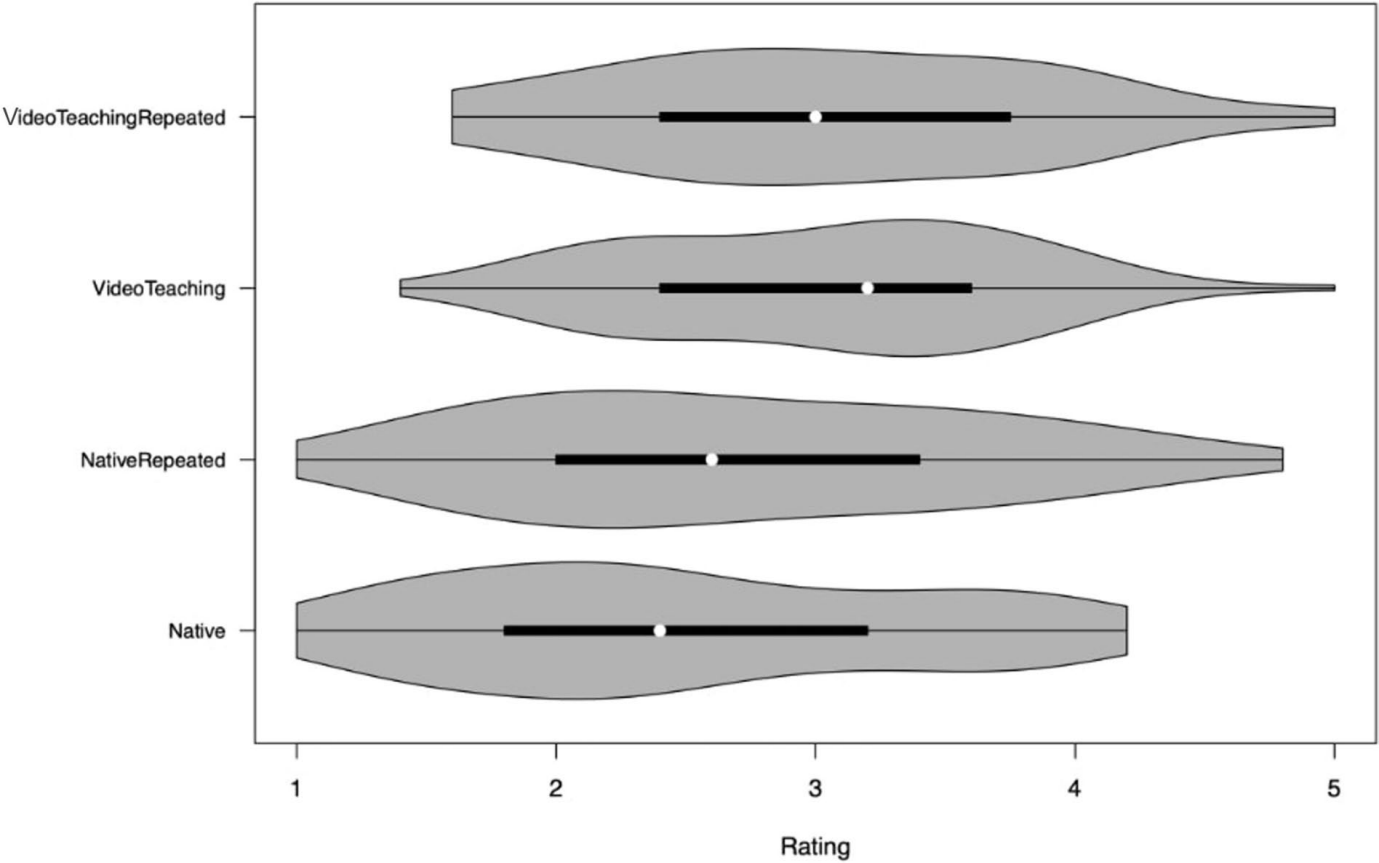
Violin plot of the GOALS Sum Score. Score/Rating distribution (1–5) in the Sum Score for GOALS across the different sequences (1–4) (q.v. Figure 2)

**Fig. 4:**
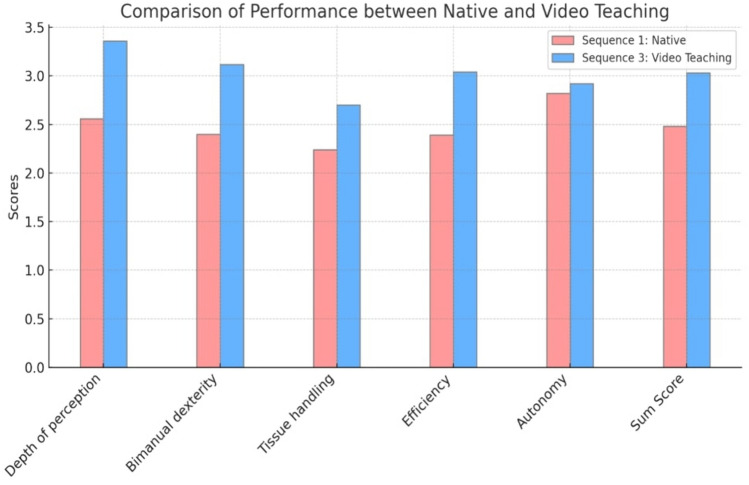
Comparison means and standard deviation in mixed-effects analysis of the effect of structured video-assisted coaching on GOALS (sub) scores in comparison to native sequence

The structured video-assisted coaching was associated with improvements in depth perception, bimanual dexterity, tissue handling, efficiency, and overall scores, with the highest improvements observed in sequences 3 and 4 according to mixed-effects model analysis (Table 1, Figs. 3, 4). However, this did not translate into a statistically significant enhancement of the candidates’ autonomy.

**Table 1:** Mixed-effects analysis of the mean and standard deviation (SD) of the effect of structured video-assisted coaching on the GOALS (sub) scores

Scale	Sequence 1: native	Sequence 2: native repeated	Sequence 3: video teaching	Sequence 4: video teaching repeated	*P* value*
Depth of perception	2.56 (0.97)	2.88 (0.91)	3.36 (0.65)	3.37 (0.80)	*P* < 0.001
Bimanual dexterity	2.40 (1.03)	2.94 (0.94)	3.12 (0.75)	3.21 (0.88)	*P* < 0.001
Tissue handling	2.24 (1.12)	2.39 (1.17)	2.70 (1.07)	2.71 (1.14)	*P* < 0.001
Efficiency	2.39 (0.95)	2.57 (1.09)	3.04 (0.81)	3.11 (0.83)	*P* = 0.037
Autonomy	2.82 (1.29)	2.82 (1.41)	2.92 (1.10)	2.81 (1.18)	*P* = 0.883
Sum score	2.48 (0.91)	2.72 (0.94)	3.03 (0.73)	3.05 (0.85)	*P* < 0.001

Mean (SD)

^*^P value from likelihood ratio tests after mixed-effect models with instructed video-assisted coaching as the fixed effect with crossed random effects for probands and reviewers. *P* values were adjusted for multiple testing to control for the false discovery rate

**Fig. 5:**
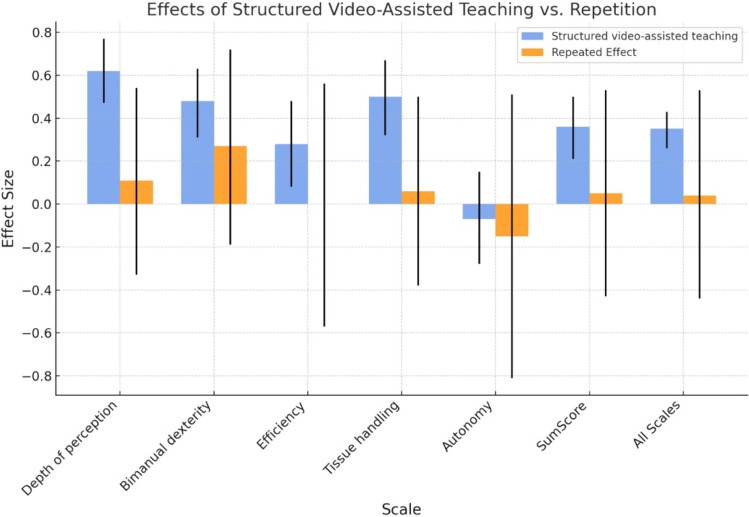
Mixed-effect analysis comparing effects of structured video-assisted teaching against repetition

When analyzing the effect of structured video-assisted coaching (sequence 3 and 4 against 1 and 2) and repetition (sequence 2 and 4 against 1 and 3) in the mixed effect-effects model, structured video-assisted coaching improved the performance among the spectrum of the skills. This positive effect ranges from 0.28 to 0.62, with the highest positive impact observed in depth of perception (0.62 95%CI: 0.47–0.77, *P* < 0.001) and the lowest impact in efficiency (0.28 95%CI: 0.08–0.48, *P* = 0.006) (Table 2, Fig. 5). No statistically significant enhancement in autonomy was observed. These results indicate that structured video-assisted coaching broadly enhances performance across most of the measured metrics. Conversely, repetition without the support of structured video-assisted coaching did not show a significant effect on these performance outcomes (Table 2, Fig. 5), suggesting that structured video-assisted coaching plays a more critical role in improving these metrics.

**Table 2:** Mixed-effect analysis comparing effects of structured video-assisted teaching against repetition

Scale	Structured video-assisted teaching effect (95% CI, *P* value)	Repeated effect (95% CI, *P* value)	Intercept effect (95% CI, *P* value)
Depth of perception	0.62 (0.47–0.77, *P* < 0.001)	0.11 (−0.33 to 0.54, *P* < 0.001)	2.64 (2.31–2.96, *P* < 0.001)
Bimanual dexterity	0.48 (0.31–0.63, *P* < 0.001)	0.27 (−0.19 to 0.72, *P* = 0.234)	2.51 (2.17–2.84, *P* < 0.001)
Efficiency	0.28 (0.08–0.48, *P* = 0.006)	−0.00 (−0.57 to 0.56, *P* = 0.998)	2.28 (1.84–2.73, *P* < 0.001)
Tissue handling	0.50 (0.32–0.67, *P* < 0.001)	0.06 (−0.38 to 0.50, *P* = 0.781)	2.44 (2.08–2.80, *P* < 0.001)
Autonomy	−0.07 (−0.28 to 0.15, *P* = 0.553)	−0.15 (−0.81 to 0.51, *P* = 0.6489)	2.84 (2.33–3.35, *P* < 0.001)
Sum Score	0.36 (0.21–0.50, *P* < 0.001)	0.05 (−0.43 to 0.53, *P* = 0.826)	2.54 (2.17–2.91, *P* < 0.001)
All scales	0.35 (0.26–0.43, *P* > 0.001)	0.04 (−0.44 to 0.53, *P* = 0.851)	2.54 (2.14–2.95, *P* < 0.001)

*P* value is significant when *P* < 0.05

Comparing all four sequences in detail against each other confirmed the association between structured video-assisted coaching with the GOALS sum score and its subscales except autonomy. Comparing sequence 1 (native) with sequence 3 (video teaching), the GOALS sum score improved by 0.49 points (95%CI: 0.26–0.73, *P* < 0.001) after structured video-assisted coaching. Sequence 4 (video teaching repeated) was superior to sequence 2 (native repeated) in terms of depth perception and tissue handling (Table 4).

Analyzing the crossed effects of structured video-assisted coaching together with demographic data and the laparoscopic experience, no association between age, gender, number of all laparoscopic operations, number of appendectomies (> 50), cholecystectomies (> 50), hernias (> 50), nor colorectal surgery (> 50) with the GOALS sum score and its sub-scores were observed. In contrast, experience in laparoscopic bariatric surgery (1–50) was associated with an increased GOALS sum score by 0.89 (95%CI: 0.30–1.48, *P* = 0.049) with a decreased effect of structured video-assisted coaching of −0.68 (95%CI: −1.00 to −0.35, *P* < 0.001). This reflects the fact that these surgeons (5 out of 27) had most likely greater experience in laparoscopic surgery.

## Discussion

The present study assessed the effect of structured video-assisted surgical coaching on the performance of experienced surgical trainees in a simulated bleeding exercise during laparoscopic surgery. The overall GOALS score as well as the depth of perception, bimanual dexterity, tissue handling, and efficiency significantly improved after structured video-assisted coaching.

Structured video-assisted coaching did not affect the autonomy, at least within the tight time boundaries of this study. This could be explained based on the definition and core of autonomy as a skill. Cassidy et al. [13] concluded that autonomy is ‘*… a pathway of progressive independence, beginning with skill development and progressing to independent decision-making with a goal of readiness for independent practice by the end of surgical training…`*.’ Autonomy incorporates the meaning and aspect of time and development. It is that aspect of time and effort in acquiring autonomy that cannot be given in the restricted timeframe of a four-day surgical course. Thus, we believe with more exercises and time also autonomy would have improved.

Control of bleeding during laparoscopic surgery is a critical skill. In this study, various severities of bleeding were simulated to assess the impact of structured video-assisted coaching on trainee performance. Results indicated a significant improvement in bleeding control skills across all four exercises following the intervention. In other studies and randomized-controlled trials video-based coaching improved suturing skills [14] and knot-tying performances in medical students [15] and skills in surgical residents [16, 17]. Comprehensive surgical coaching enhanced surgical skills in the operating room for laparoscopic jejunostomy [18]. In all these published studies, independently of the tasks given, or the level of experience (students and residents) coaching improved performances of GOALS or OSATS scores [9, 19]. However, one-to-one coaching is time consuming and costly. Structured video-assisted coaching is a valuable addition to the traditional surgical training and could offer enormous advantages to the economics of Health Systems [20]. In the era of personalization, demand for proficiency, evidence-based surgical performance, standardization, safety, and cost effectiveness, modernizing surgical training is essential.

In this study, the candidates were not only asked to perform a well-defined task, e.g., knotting but also experienced the psychological pressure of the acute/emergency problem. They had to identify and localize the bleeding, prepare the site (suction, compression), and control the problem (clip or suture). To our knowledge, this is the first study training a complication (bleeding) of laparoscopic surgery. Future training curricula need to incorporate also training in complication management.

The study has some limitations. The sample size was small and the time frame was short. In addition, the effect of the intervention (the coaching) could not be fully analyzed as we did not include a control group. Due to the nature of the course and the tight schedule, a longer study duration and a control group were not possible. Additionally, self-reporting of previous surgical experience always has a possible risk of bias [21]. However, skills were assessed blinded by validated tools and each individual trainee progressed. Finally, the question arises whether the statistically significant differences in the GOALS scores for ‘depth of perception,’ ‘bimanual dexterity,’ and ‘tissue handling’ have a clear clinical relevance for practice. Our study cannot conclusively assess this, but we are convinced that structured coaching steepens the learning curve during training and that the participants benefit considerably as a result. Ultimately, further follow-up studies are needed to substantiate the effect shown here.

## Conclusion

The use of structured video-assisted coaching in training young surgeons improved surgical performance in complex, stress-eluting tasks like laparoscopic bleeding control. Improvements were observed in various skills in a short time. The findings of this study support the incorporation of structured video-assisted coaching and complication management exercises into surgical training curricula.

## Appendix

See Tables 3 and 4.

**Table 3:** Demographics & laparoscopic experience

Variable	Label	Total *N* = 27
Age	Mean (SD)	36.4 (4.7)
	Valid *N*	25 (93%)
Gender	Male	14 (52%)
	Female	12 (47%)
	k.a	1 (1%)
Operation total	mean(SD)	280 (147)
Appendectomy	< 20	1 (4%)
	20–50	5 (19%)
	51–100	12 (44%)
	> 100	9 (33%)
Cholecystectomy	20–50	4 (15%)
	51–100	12 (45%)
	> 100	11 (40%)
Colorectal	0	2 (7%)
	< 20	17 (63%)
	20–50	3 (11%)
	51–100	3 (11%)
	> 100	2 (8%)
Bariatrics	0	22 (82%)
	< 20	3 (11%)
	20–50	2 (7%)
Hernia	0	4 (15%)
	< 20	4 (15%)
	20–50	6 (22%)
	51–100	9 (33%)
	> 100	4 (15%)

**Table 4:** Treatment Tukey contrasts for GOALS scores

Scale	Sequence	Treatment	Effect (95% CI, *P* value)
Depth perception	3-1	Video Teaching—Native	0.80 (0.54–1.05, *P* < 0.001)
	4–2	Video Teaching Repeated—Native Repeated	0.39 (0.10–0.68, *P* = 0.004)
Bimanual dexterity	3-1	Video Teaching—Native	0.73 (0.45–1.00, *P* < 0.001)
	4-2	Video Teaching Repeated—Native Repeated	0.16 (0.16–0.47, *P* = 0.565)
Efficiency	3-1	Video Teaching—Native	0.37 (0.03–0.70, *P* = 0.027)
	4-2	Video Teaching Repeated—Native Repeated	0.17 (0.22–0.55, *P* = 0.676)
Tissue handling	3–1	Video Teaching—Native	0.61 (0.32–0.90, *P* < 0.001)
	4-2	Video Teaching Repeated—Native Repeated	0.35 (0.02–0.68, *P* = 0.033)
Autonomy	3–1	Video Teaching—Native	−0.01 (0.38–0.35, *P* = 1.000)
	4-2	Video Teaching Repeated—Native Repeated	−0.13 (0.55–0.28, *P* = 0.834)
Sum Score	3-1	Video Teaching—Native	0.49 (0.26–0.73, *P* < 0.001)
	4–2	Video Teaching Repeated—Native Repeated	0.17 (0.09–0.44, *P* = 0.334)

Mixed-effects regression with reviewer and proband as random effects and Tukey contrasts to compare the effects of the 4 sequences
